# Mass Spectra of Some Deuteroethanes

**DOI:** 10.6028/jres.065A.010

**Published:** 1961-04-01

**Authors:** Edith I. Quinn, Fred L. Mohler

## Abstract

Mass spectra of seven of the nine possible deuteroethanes have been measured. When the patterns are computed on a scale to make the sums of ions equal, it is found that the abundances of molecule ions differ nearly twofold in the different molecules, and there are large differences in the patterns of the two C_2_H_4_D_2_ molecules. The peaks in the C_1_ range are compared with the patterns predicted for simple breaking of the C—C bond and dissociation of the methyl radicals. The “weighting factors” for removing H and D, HD, and 2D, etc., from C_2_HD_5_ are computed and also the weighting factors for H^+^ and D^+^ for all the deuteroethanes are given.

## 1. Introduction

While there is a rather extensive literature on mass spectra of deuterohydrocarbons, published data on deuteroethanes are fragmentary. Schissler, Thompson, and Turkevich [1]^3^ published mass spectra of four deuteroethanes in 1951 in the mass range 12 to 36 and Dibeler [2] published a mass spectrum of C_2_D_6_ of good purity.

We were able to obtain samples of seven of the nine possible deuteroethanes. Two of these were synthesized by M. deHemptinne of the University of Louvain and the others were purchased from Merck Inc. Mass spectra were recorded with a 180° Consolidated model 21–103 mass spectrometer following standard operating procedures. The ionizing voltage is 70 v except as noted below.

## 2. Results

The samples were all of good chemical purity and an isotopic analysis was made by recording the spectrum at an ionizing voltage low enough to give molecule ions but not fragment ions [3]. Table 1 lists the seven deuteroethanes, the isotopic analysis and the source of the samples. This is a difficult analysis for ethane as the appearance potentials of the fragment ions are close to the ionization potential of the molecule. Field and Franklin [4] give appearance potentials as follows: $C_{2}H_{6}^{+}$, 11.6 ev, C_2_H_5_^+^, 12.8 ev; and C_2_H_4_^+^, 12.1 ev. This makes the measurement insensitive. (The impurity with one less D atom than the sample may be uncertain by 10 percent of the stated amount.) The mole percent impurities listed are consistent with the purity reported by Merck in round numbers as 98 and 99 atom percent.

Table 2 gives the mass spectra of C_2_H_6_ and the seven deuteroethanes. The contributions of ions containing C^13^ and of isotopic impurities from table 1 have been subtracted from the spectra. The patterns of the different molecules have been normalized in an unconventional manner. It seemed logical to present the spectra on a scale to make the parent ion abundances all equal but when this is done it is found that there is a wide variation in the sums of all the ions in the spectrum. It seems unlikely that there is a wide variation in the total ionization of molecules that differ only in the isotopic masses of the atoms although difference of mass distribution will change the pattern of dissociation. In table 2 the patterns are on a scale to make the sums of the ions equal to the sum for C_2_H_6_ with the conventional scale of abundances used for the C_2_H_6_ pattern.

It is evident that on this scale there is a wide variation of the abundances of the molecule ions. It ranges from 13.0 for CH_3_·CD_3_ to 23.0 for C_2_H_5_D. Both of the highly unsymmetrical molecules CH_3_·CD_3_ and CH_3_·CHD_2_ give low values of the molecule ion. There are many other conspicuous differences between the mass spectra of the symmetrical and unsymmetrical molecules of C_2_H_4_D_2_ in columns 4 and 5.

As a general rule the removal of D atoms from deuterohydrocarbons is less probable than the removal of H but C_2_D_6_ affords a notable exception. The C_2_D_4_^+^ ion has an abundance of 113.5 as compared with 100 for C_2_H_4_^+^ of C_2_H_6_. In the patterns of ethanes containing H and D two different ions contribute to many of the peaks. Thus C_2_H_2_^+^ and C_2_D^+^ contribute to mass 26, C_2_H_3_^+^ and C_2_HD^+^ to 27 etc. In the deuteroethane spectra there is no safe basis for computing the relative probability of removing H and D or several H and D atoms except in the C_2_HD_5_ spectrum. In that spectrum each peak comes from a single isotopic fragment ion.

Table 3 gives in the third column the pattern of C_2_HD_5_ with the peaks grouped in pairs of ions containing 1, 2, 3, etc., hydrogenic atoms. The sums of these pairs in column 4 give the relative probability of removing one to five atoms. This times the a’priori probability gives, in column 6, the pattern predicted if removal of H and D were random. The experimental values divided by the theoretical give the “weighting factors” for removing H and D atoms and all possible combinations of these. It is seen that except for the 25, 26 pair, ions involving loss of the H atom have a weight factor greater than one and the others less than one. This is qualitatively consistent with experiments with other deuterohydrocarbons. The weighting factors for removing H and D, 1.67 and 0.86 are of the magnitudes found for CHD_3_ [5] and C_2_HD_3_ [6]. There is, however, no regular trend in weighting factors for removing 1, 2, 3, etc., atoms from C_2_HD_5_ such as was found in the deuteroethylene spectra [6].

In the last column of table 3 the observed pattern of C_2_D_6_ is given and it is quite similar to the “sums of pairs” of C_2_HD_5_ in column 4. It is not surprising that C_2_HD_5_ is more like C_2_D_6_ than C_2_H_6_.

The ions in the mass 12 to 18 range (table 2) involve breaking the C—C bond and if this bond breaks without rearrangement of H and D atoms there will be a simple relation between patterns of unsymmetrical molecules and patterns of two symmetrical molecules. Thus the CH_3_·CD_3_ fragment ions in the C_1_ range will involve contributions from CH_3_ and from CD_3_ as given by the C_2_H_6_ and C_2_D_6_ patterns. However the observed pattern shows a small 17 peak from CHD_2_^+^ so there is some rearrangement.

Table 4 compares the observed patterns for unsymmetrical molecules in the C_1_ range and the patterns computed from the spectra of two symmetrical molecules. The last two rows give the basis of computation. Thus the CH_3_·CH_2_D pattern is compared with 0.59 times the patterns of CH_2_D·CH_2_D plus 0.53 times the pattern of C_2_H_6_. In some cases as in the CHD_2_·CD_3_ spectrum there is fairly good agreement between the observed and computed spectra while in CH_3_·CHD_2_ the agreement is poor. In the CH_3_·CD_3_ pattern there is a peak at 17 that comes from the rearrangement giving CHD_2_^+^ and CH_2_D and the residual on the 16 peak comes from the same rearrangement with the charge on CH_2_D.

Table 5 gives the observed H^+^ and D^+^ ions and weighting factors for H^+^ and D^+^. A small but rather uncertain correction for H_2_^+^ has been made to the mass 2 peaks of table 2. The weighting factor is computed from the observed value divided by H^+^+D^+^) times the apriori probability. In C_2_D_6_ it is simply the ratio of D^+^ of C_2_D_6_ to H^+^ of C_2_H_6_. There is a progressive increase in the weighting factor for H^+^ as the number of D atoms in the atom increases. The factors for D^+^ do not change progressively.

Table 2 includes some small half integer peaks that come from doubly charged ions of mass 27, 29, 31, and 33. Doubly charged ions of even mass number are masked by singly charged ions. The more abundant ions contain 4 and 5 hydrogenic atoms. In the case of C_2_HD_5_ the relative abundance of the pairs of ions containing 4 or 5 hydrogenic atoms can be computed if it is assumed that the same weighting factors apply in doubly and singly charged ions. Peak 15.5 of abundance 0.57 comes from C_2_HD_3_^++^ and from table 3, C_2_D_4_^++^ will have an abundance of about 0.39. The abundance of the pair is 0.96. The 16.5 peak of abundance 0.24 is C_2_HD_4_^++^. C_2_D_5_^++^ is about 0.09 and the abundance of the pair of ions with 5 hydrogenic ions is 0.33. The abundance of C_2_H_5_^++^ of C_2_H_6_ is 0.54.

The doubly charged ions of C_2_HD_5_ make contributions of about 0.39 to the 16 peak and 0.09 to the 17 peak on the basis of the above assumptions. There is no good basis for computing contributions of doubly charged ions to the 14, 15, 16, and 17 peaks of other deuteroethanes but they are a source of experimental uncertainty in the data of table 4. In the case of C_2_H_6_ the abundance of C_2_H_4_^++^ will be approximately like that of the pair of ions from C_2_HD_5_ of abundance 0.96.

## 3. Summary

The mass spectra of deuteroethylenes [6] and deuteromethanes [5] show a regular trend in the probability of removing H and D atoms from molecules with increasing numbers of D atoms in the molecule. In the C_2_ range there is no appreciable difference between the patterns of symmetrical and unsymmetrical dideuteroethylenes. In these molecules it is logical to compute “weighting factors” on the basis of the C_2_H_4_ or CH_4_ spectra. In the case of the ethanes there is no regular trend. There are large differences in the relative abundance of molecule ions and there are striking differences between symmetrical and unsymmetrical molecules. Only in the case of the C_2_HD_5_ pattern is there a logical basis for computing weighting factors for removing H and D.

It is consistent with the statistical theory of mass spectra of polyatomic molecules [7] that there will be some differences in patterns of deuteroethanes because of differences in vibration levels of D and H atoms. This is a complicated problem and beyond the scope of this paper.

It will be of interest to obtain the patterns of the other deuteroethanes, CH_2_D·CHD_2_ and CH_2_D·CD_3_ for these undoubtedly differ from the isomers available for this study. This is needed for analytical purposes. For instance there is a small uncertainty in this work because C_2_H_2_D_4_ is the most abundant impurity in C_2_HD_5_ and C_2_H_3_D_3_ is the most abundant in CHD_2_·CHD_2_. We used the patterns of CHD_2_·CHD_2_ and CH_3_CD_3_ for the corrections but it would be better to use the mean patterns of the two isomers if both were known.

The experimental material in this paper was used by Miss Quinn as a Master’s thesis at the University of Alabama.

## Figures and Tables

**Table 1:** Mole percent impurities in deuteroethanes

Sample	Impurity	Mole percent	Source
			
C_2_H_5_D	$\{\begin{array}{l} C_{2}H_{6}\\ C_{2}H_{4}D_{2} \end{array}$	3.6 1.4	} deHemptinne.
CHD_2_·CH_3_	C_2_H_5_D	3.2	Merck Inc.
CH_2_D·CH_2_D	$\{\begin{array}{l} C_{2}H_{5}D\\ C_{2}H_{3}D_{3} \end{array}$	1.2 .3	} Merck Inc.
CH_3_·CD_3_	C_2_H_4_D_2_	5.0	Merck Inc.
CHD_2_·CHD_2_	$\{\begin{array}{l} C_{2}H_{3}D_{3}\\ C_{2}{\text{HD}}_{5} \end{array}$	2.7 .2	} Merck Inc.
C_2_HD_5_	$\{\begin{array}{l} \begin{array}{l} C_{2}H_{2}D_{4}\\ C_{2}D_{6} \end{array} \end{array}$	6.2 .4	} deHemptinne.
C_2_D_6_	C_2_HD_5_	4.0	Merck Inc.

**Table 2:** Mass spectra of deuteroethanes on a scale to make the sums of ions equal

M/e	C_2_H_6_	C_2_H_5_D	CH_2_D-CH_2_D	CHD_2_-CH_3_	CH_3_CD_3_	CHD_2_-CHD_2_	C_2_HD_5_	C_2_D_6_
								
1	4.11	5.04	3.98	4.63	3.72	2.26	1.13	…………
2	.46	.84	.92	.95	1.24	1.56	1.98	2.61
3	…………	.12	.27	.13	…………	.22	.11	…………
4	…………	…………	.04	.03	…………	.07	.14	.18
12	.90	1.02	.95	1.39	1.31	1.03	1.18	1.16
13	1.74	1.55	1.04	1.92	1.41	.39	.27	…………
14	4.12	2.26	2.07	3.60	2.96	1.86	2.32	2.60
15	5.87	5.12	3.41	3.16	3.17	2.28	1.12	…………
16	…………	3.19	5.43	1.19	1.87	2.63	3.53	5.63
17	…………	…………	.32	1.84	.88	5.60	2.79	…………
18	…………	…………	…………	…………	1.69	.26	3.08	6.21
24	1.09	1.00	.92	1.63	1.33	.82	.76	.73
25	5.16	4.00	2.36	4.71	2.66	.84	.89	…………
26	26.2	13.4	9.03	21.1	12.0	3.97	3.71	4.02
27	36.8	24.3	17.1	29.5	19.6	10.0	5.39	…………
28	100	42.3	32.0	43.1	28.6	18.3	20.3	24.1
29	19.3	84.1	54.6	49.4	41.8	19.0	12.4	…………
30	22.1	15.9	59.8	35.1	66.0	45.5	19.6	31.2
31	…………	23.0	14.3	10.5	14.0	58.2	66.8	…………
32	…………	…………	19.1	13.5	10.6	26.5	45.7	113.5
33	…………	…………	…………	…………	13.0	8.7	12.4	…………
34	…………	…………	…………	…………	…………	18.0	4.79	17.1
35	…………	…………	…………	…………	…………	…………	17.4	…………
36	…………	…………	…………	…………	…………	…………	…………	18.7
13.5	.06	.09	…………	.09	.04	…………	…………	…………
14.5	.54	.84	.47	.35	.26	.03	…………	…………
15.5	…………	.01	.36	.24	.37	.59	.57	…………
16.5	…………	…………	…………	…………	…………	.20	.24	…………

**Table 3:** Pattern of *C_2_HD_5_* and theoretical pattern in C_2_ range

M/e	Ions	Pattern	Sums of^a^ pairs	a’priori probability	Theoretical^b^	Weight^c^ factor	C_2_D_6_ pattern
							
24	C_2_	0.76	(0.76)	1	0.76	…………	0.73
25	C_2_H	.89	…………	⅙	.77	1.15	…………
26	C_2_D	3.71	4.60	⅚	3.83	.97	4.02
27	C_2_HD	5.39	…………	⅓	8.57	.63	…………
28	C_2_D_2_	20.3	25.7	⅔	7.13	1.18	24.1
29	C_2_HD_2_	12.4	…………	½	16.0	.78	…………
30	C_2_D_3_	19.6	32.0	½	16.0	1.22	31.2
31	C_2_HD_3_	66.8	…………	⅔	75.	.89	…………
32	C_2_D_4_	45.7	112.5	⅓	37.5	1.22	113.5
33	C_2_HD_4_	12.4	…………	⅚	14.3	.86	…………
34	C_2_D_5_	4.79	17.2	⅙	2.87	1.67	17.1
35	C_2_HD_5_	17.4	(17.4)	1	17.4	1.00	…………
…………	…………	…………	…………	…………	…………	C_2_D_6_	18.7

a The sums of the indicated pairs give the relative abundance of ions with 1 to 5 hydrogenic atoms.

b This column gives the a’priori probability, times the abundances of column 4.

c This gives the ratio of the observed pattern and the theoretical pattern.

**Table 4:** Mass spectra in the *C_1_* range and computed values^a^

M/e	C_2_H_5_D		CH_3_·CHD_2_		CH_3_·CD_3_		C_2_HD_2_·CD_3_	
	exp.	comp.	exp.	comp.	exp.	comp.	exp.	comp.
								
12	1.02	1.04	1.39	0.71	1.31	0.81	1.18	1.06
13	1.55	1.54	1.92	.85	1.41	.94	.27	.19
14	2.26	3.41	3.60	2.31	2.96	2.94	2.32	2.16
15	5.12	5.12	3.16	3.16	3.17	3.17	1.12	1.13
16	3.19	3.19	1.19	.87	1.87	1.53	3.53	3.99
17	…………	(.19)	1.84	1.84	.88	…………	2.79	2.79
18	…………	…………	…………	(.09)	1.69	1.69	3.08	3.08
				
Comp. from	.59×CH_2_D		.33×CHD_2_		.272×CD_3_		.475×CD_3_	
	.53×CH_3_		.41×CH_3_		.540×CH_3_		.50×CHD_2_	

a Mass spectra of unsymmetrical molecules are computed from symmetrical molecules on the assumption that these ions come from breaking of the C—C bond without rearrangement of H atoms.

**Table 5:** Hydrogenic ions

	C_2_H_6_	C_2_H_5_D	CH_2_D CH_2_D	CHD_2_-CH_3_	CH_3_-CD_3_	CHD_2_-CHD_2_	C_2_HD_5_	C_2_D_6_
								
H^+^	4.11	5.04	3.98	4.63	3.72	2.26	1.13	…………
D^+^	…………	.60	.72	.85	1.24	1.53	1.98	2.61
Weight Factor								
For H	…………	1.07	1.27	1.26	1.50	1.79	2.17	…………
For D	…………	.64	.46	.46	.50	.61	.76	(.63)

## Footnotes

3 Figures in brackets indicate the literature references at the end of this paper.
